# The Unmet Needs of Pancreatic Cancer Carers Are Associated with Anxiety and Depression in Patients and Carers

**DOI:** 10.3390/cancers15225307

**Published:** 2023-11-07

**Authors:** Thi N. T. Huynh, Gunter Hartel, Monika Janda, David Wyld, Neil Merrett, Helen Gooden, Rachel E. Neale, Vanessa L. Beesley

**Affiliations:** 1 Population Health Department, QIMR Berghofer Medical Research Institute, Brisbane, QLD 4006, Australia; justine.huynh@qimrberghofer.edu.au (T.N.T.H.); gunter.hartel@qimrberghofer.edu.au (G.H.); rachel.neale@qimrberghofer.edu.au (R.E.N.); 2 Faculty of Medicine, The University of Queensland, Brisbane, QLD 4006, Australia; m.janda@uq.edu.au (M.J.); david.wyld@health.qld.gov.au (D.W.); 3 School of Nursing, Queensland University of Technology, Brisbane, QLD 4059, Australia; 4 Cancer Care Services, Royal Brisbane and Women’s Hospital, Herston, QLD 4029, Australia; 5 School of Medicine, Western Sydney University, Sydney, NSW 2560, Australia; neil.merrett@health.nsw.gov.au; 6 School of Nursing and Midwifery, University of Sydney, Sydney, NSW 2006, Australia; hmg@iinet.net.au

**Keywords:** pancreatic cancer, family carers, supportive care needs, anxiety, depression

## Abstract

**Simple Summary:**

This is the first population-based study to quantitatively assess the supportive care needs of pancreatic cancer patients’ carers and their associations with psychological outcomes for patient–carer dyads. We show that for this patient group, carers have exceptionally high levels of unmet needs, particularly in the healthcare and information domains, and some of these needs are strongly associated with anxiety and depression among carers and the patients they care for. The results of this study provide a strong rationale for the implementation of optimal care guidelines to promote early referrals to palliative care for pancreatic cancer patients in which the routine assessment and management of supportive care needs among family carers (and patients) occurs.

**Abstract:**

Pancreatic cancer has one of the lowest survival rates, and patients experience debilitating symptoms. Family carers provide essential daily care. This study determined the prevalence of and risk factors for unmet supportive care needs among carers for pancreatic cancer patients and examined which carer needs were associated with anxiety and depression in carers and patients. Eighty-four pancreatic cancer patients and their carers were recruited. The carers completed a needs survey (SCNS-P&C). Both carers and patients completed the Hospital Anxiety and Depression Scale. Log binomial regression was used to identify associations between carer needs and anxiety and depression among carers and patients. The top 10 moderate-to-high unmet needs reported by ≥28% of carers were related to healthcare (e.g., discussing concerns with doctors) and information need domains (e.g., information about a patient’s physical needs), plus one other item related to hospital parking. Being male or caring for a patient within 4 months of their diagnosis were associated with greater unmet needs. Some unmet needs, including ‘accessing information about treatments’ and ‘being involved in patient care’, were associated with both carers and patients having anxiety and depression. Carers should be involved in health care consultations and provided with information and opportunities to discuss concerns.

## 1. Introduction

Pancreatic cancer it is the third most common cause of cancer death in developed countries [1]. Surgical resection is the only potentially curative treatment [2]. This cancer is typically asymptomatic early in its development, and there is no reliable screening test for early detection; thus, approximately 80% of patients present with unresectable (metastatic or locally advanced) disease [3]. During the limited time prior to death (the median survival time is 5 months [4]), patients suffer from fatigue, jaundice, abdominal or back pain, poor appetite, unintended weight loss, nausea, vomiting [5], anxiety and depression [6]. Supportive care plays an important role in managing these symptoms.

In the pancreatic cancer setting, family carers provide considerable care alongside professionally trained carers. They manage symptoms or treatment-related side effects and help with daily activities and end-of-life decision making [7]. They also provide financial, emotional, social and spiritual support [8] while facing the impending loss of their loved one. When caring demands exceed their resources, they can experience negative impacts on their mental health [8]. A systematic review found that 33% of people providing informal care to patients with pancreatic cancer have symptoms indicative of clinical anxiety and 12–32% have symptoms indicative of clinical depression [9].

Understanding and satisfying carers‘ explicit supportive care needs can result in cost savings for health systems [10]. There are no published quantitative studies documenting the supportive care needs of family carers for people diagnosed with pancreatic cancer [9]. The complex symptoms and the rare and rapidly progressing nature of pancreatic cancer are likely to be uniquely challenging for these carers. The aims of this work were to determine the prevalence of and risk factors for the unmet supportive care needs of this population, using quantitative methods and a validated multidimensional needs assessment tool. Additionally, we examined which specific carer needs were correlated with carers’ and patients’ anxiety or depression.

## 2. Materials and Methods

### 2.1. Participants and Procedures

Participants were recruited from the Queensland Pancreatic Cancer Study—Quality of Life (QPCS-QoL), which was a longitudinal, repeated-measures study of patient–carer dyads who self-reported outcomes at two, four, six and eight months after a diagnosis of pancreatic cancer. The QoL study was nested within the QPCS, a population-based case–control study examining environmental and genetic risk factors for pancreatic cancer [6]. The QPCS used a rapid ascertainment approach, recruiting patients diagnosed with pancreatic cancer between January 2007 and June 2011, as early as possible after their diagnosis, based on referral from a state-wide network of treating clinicians in hospitals and private practices. 

QPCS participants recruited from July 2009 were invited to participate in the QPCS-QoL and to nominate a carer who was also invited to participate in this sub-study. Of the 351 QPCS participants recruited from July 2009, 97 were excluded due to a physical or mental inability to complete the study documents, 57 refused, 23 died shortly after receiving the questionnaire, and 38 patients did not return the questionnaire. One hundred thirty-six QPCS participants completed at least one QoL questionnaire (54% of those approached). Of these 136 patients, 21 did not nominate a carer and 31 carers declined to participate. Thus, 84 (62%) patient–carer dyads completed the questionnaire(s). We used data from the first questionnaire completed by the 84 dyads in this analysis.

### 2.2. Measures

#### 2.2.1. Personal and Disease Information

We collected the age, gender and education level of the patients and carers. Carers were also asked about their relationship to patients and if they were living together. Postcodes were used to assign the remoteness of participants’ residences based on the Accessibility and Remoteness Index of Australia [11]. 

Clinical information about the patients’ date of diagnosis and disease status, defined as resectable, locally advanced or metastatic, was extracted from medical records. 

#### 2.2.2. Carers’ Supportive Care Needs

The Supportive Care Needs Survey—Partners and Caregivers (SCNS-P&C) was used to assess 45 need items across four domains: healthcare service (10 items), psychological and emotional (14 items), work and social (7 items) and information (8 items) [12]. It also assesses six other items that are not classified into a domain. The SCNS-P&C asks participants to rate each need item across five response categories: ‘1 = no unmet need—not applicable’, ‘2 = no unmet need—satisfied’, ‘3 = low unmet need’, ‘4 = moderate unmet need’ and ‘5 = high unmet need’. In this analysis, we dichotomised each item into ‘no-or-low need’ versus ‘moderate-to-high need’. We also calculated the number of moderate-to-high unmet needs in each domain and overall. The domains have been shown to have good internal validity; Cronbach’s alpha ranged from 0.88 to 0.94 [12]. 

#### 2.2.3. Carers’ and Patients’ Anxiety and Depression

Anxiety and depression symptoms were assessed using the Hospital Anxiety and Depression Scale (HADS) [13]. It comprises two separate 7-item subscales. The HADS-A measures symptoms of anxiety, including restlessness, nervousness and feelings of panic. The items assess emotional and cognitive aspects of anxiety, such as feeling tense or having a sense of impending doom. The HADS-D assesses symptoms of depression, including low mood, loss of interest or pleasure and a sense of hopelessness. It explores emotional and cognitive aspects of depression, like feeling sad and experiencing a lack of enjoyment in daily activities. Participants rate each symptom as ‘0 = absent’; ‘1 = occasional’; ‘2 = quite often’; or ‘3 = very often’. The total score of each subscale ranges from 0 to 21 and is classified as normal (0–7), sub-clinical (8–10) or clinical (11–21). The HADS is a reliable measure of anxiety and depression, with respective Cronbach’s alpha values of 0.88 and 0.92 for internal validity [14], and has good external validity with the Depression Anxiety Stress Scales (DASS-42) (Pearson’s correlation coefficient r = 0.76 (*p* < 0.001)) [15]. 

### 2.3. Statistical Analysis

To identify the carers’ most prevalent unmet needs, we calculated the proportion reporting each need as moderate-to-high out of the number of participants who completed each item. The amount of missing data for each need item varied from 2 to 7 carer responses except for the need item ‘reducing stress in the person with cancer’s life’. This item had 60 missing responses due to being inadvertently deleted from an updated version of the questionnaire. As a consequence, this variable is not reported in this manuscript.

To identify which personal and disease risk factors were associated with carers reporting at least one moderate-to-high need, we used log-binomial regression to estimate prevalence ratios (PRs) and 95% confidence intervals (CIs). We considered PRs ≥ 2.0 to be clinically significant. 

Log-binomial regression models were also used to estimate associations between (a) carers’ individual need items, (b) the number of needs reported (categorised into tertiles), (c) having a need in each domain and (d) the number of domains carers had a need in (categorised as 0, 1–3 or 4) and (i) the carers’ sub-clinical or clinical anxiety, (ii) the carers’ sub-clinical or clinical depression, (iii) the patients’ sub-clinical or clinical anxiety and (iv) the patients’ sub-clinical or clinical depression. The Expectation Maximisation algorithm was applied to handle missing data. This method uses observed data to obtain maximum likelihood estimates of variables when missing values are present [16]. When the log-binomial model did not converge, we used Poisson regression models [17].

All analyses were conducted using R software version 4.1.2 (R Foundation for Statistical Computing, Vienna, Austria).

## 3. Results

### 3.1. Sample Characteristics

The characteristics of the 84 patient–carer dyads are shown in Table 1. In most cases, spouses or partners (81%) took on the role of informal carer, and almost all carers (94%) lived with the patient. Nearly three-quarters of the carers were female, 62% were 60 years of age or older and for 57%, the highest level of education attained was a high school diploma or lower. The majority of patients were male (66%) and 60 years of age or older (74%), and less than half completed a high school education (44%). Thirty-six percent of patients were treated via surgical resection. Of the 54 patients who did not undergo resection, more than half (30; 56%) had metastatic disease. At the time of the questionnaire’s completion, all patients were between 0and 9 months from the time of the pancreatic cancer diagnosis; 35% were within 2 months and 82% were within 4 months.

### 3.2. The Top Moderate-to-High Unmet Supportive Care Needs of Carers

Overall, 63% of the carers reported having at least one moderate-to-high unmet need. The top 10 needs were from the healthcare services (five items) and information (four items) supportive care domains, plus one non-specific item (Table 2). More than a third of carers reported a moderate-to-high unmet need for information about the person with cancer’s physical needs (39%), opportunities to discuss concerns with doctors (36%), accessible hospital parking (36%), help with fear about the patients’ deterioration (35%) and information about the benefits and side-effects of treatments (34%). The most frequently reported psychological needs were for help with balancing the needs of the person with cancer and their own needs (26%), understanding the patient experience (23%), finding meaning (21%) and making decisions in the context of uncertainty (21%). The most frequently unmet work/social needs were for help with the impact caring has had on their working life or usual activities (24%) and adapting to changes in the patient’s work or activities (23%).

### 3.3. Personal and Disease Risk Factors Associated with Carers Having at Least One Moderate-to-High Unmet Need

While the numbers in some groups were small, bivariable analyses indicated that male carers were more likely than female carers to report a moderate-to-high unmet need (PR = 1.36; 95% CI = 1.01–1.85) and, compared to carers for patients who were 5–9 months post-diagnosis, those within 2 or 3–4 months of the diagnosis had significantly higher prevalences of moderate-to-high unmet needs (PR = 2.46; 95% CI = 1.02–5.92 and PR = 2.81; 95% CI = 1.19–6.63, respectively) (Table 3). The carer’s age, relationship to the patient and level of education and the patient’s disease status were not significantly associated with carers having an unmet need (Table 3).

### 3.4. Relationship between Carers’ Unmet Needs and Carers’ Anxiety and Depression

There was a significant association between carers reporting moderate-to-high unmet need(s) in any of the supportive care domains and also reporting subclinical or clinical anxiety and depression (Table 4, Supplementary Tables S1 and S2). The strongest associations were between depression and the healthcare (PR 5.08; 95% CI 2.12–12.20) or psychological needs domains (PR 3.85; 95% CI 1.72–8.61).

Carers reporting unmet needs in four domains were more than three times as likely to have anxiety (PR 3.57; 95% CI 1.90–6.70) and five times as likely to have depression (PR 5.10; 95% CI 1.92–13.52) compared to carers not reporting any unmet needs in any domain. Carers reporting unmet needs in 1–3 domains only showed a significant association with carer anxiety (PR 2.72; 95% CI 1.39–5.33). Compared with carers who had no moderate-to-high needs, carers with 13–35 moderate-to-high needs (the highest tertile) were significantly more likely to have anxiety and depression (PR 3.32 and 95% CI 1.67–6.60 and PR 4.43 and 95% CI 1.68–11.67, respectively).

The majority (35) of the 44 individual need items reported by carers were significantly associated with both carer anxiety and depression (Supplementary Table S3). The strongest individual needs associated with carer anxiety were needing help finding meaning in the patient’s illness (PR 2.32; 95% CI 1.67–3.22), decision making in uncertainty (PR 2.22; 95% CI 1.57–3.15) and needing information about the patient’s prognosis (PR 2.19; 95% CI 1.53–3.13). The strongest items associated with carer depression were needing to talk to other cancer carers (PR 4.50; 95% CI 2.92–6.93), needing help with knowing how to discuss cancer socially (PR 3.67; 95% CI 2.55–5.28) and needing help with feelings about death (PR 3.46; 95% CI 1.98–6.03). 

### 3.5. Relationship between Carers’ Unmet Needs and Patients’ Anxiety and Depression

Carers who reported at least one moderate-to-high need in the healthcare, psychological or work/social domains were significantly more likely to be caring for a patient with anxiety or depression symptoms. The strongest association was between carers having at least one unmet psychological need and patient anxiety (PR 4.38; 95% CI 1.81–10.64). Carers reporting at least one moderate-to-high information need was associated with patient anxiety (PR 3.29; 95% CI 1.36–8.00) but not with patient depression. 

Carers who reported moderate-to-high needs across all four domains were four times (PR 4.42; 95% CI 1.63–11.95) more likely to be caring for a patient with anxiety symptoms and two times (PR 2.21; 95% CI 1.08–4.51) more likely to be caring for a patient with depressive symptoms compared to carers who did not report any moderate-to-high needs. An increasing number of moderate-to-high unmet needs reported by carers was associated with an increased likelihood of patients experiencing anxiety or depression. Compared with patients whose carers had no moderate-to-high needs (the lowest tertile), patients whose carers reported 13–35 moderate-to-high needs (the highest tertile) were significantly more likely to have anxiety and depression (PR 4.80; 95% CI 1.52–15.10 and PR 2.40; 95% CI 1.06–5.45, respectively).

Having high supportive care needs in each of 14 of the 44 individual need items was significantly associated with patient anxiety, and 11 items were significantly associated with patient depression (Supplementary Table S3). The strongest associations with anxiety and depression in cancer patients occurred with carers having a need for help with adjusting to changes in the patient’s body (PR 3.03 and 95% CI 1.65–5.56 and PR = 2.95 and 95% CI 1.72–5.06, respectively). Balancing their own and the patient’s needs (PR 2.93; 95% CI 1.52–5.63) and obtaining the best medical care for patients (PR 2.59; 95% CI 1.32–5.09) were the other carer unmet needs strongly associated with patient anxiety. Carers needing help with communicating with the family (PR 2.46; 95% CI 1.41–4.28) and practical caring tasks (PR 2.27; 95% CI 1.17–4.42) were significantly more likely to be caring for a patient with depression.

There were five unmet carer needs significantly associated with all psychological outcomes for carers and patients. These included needing help with ‘accessing information about the benefits and side-effects of treatments’, ‘obtaining the best medical care for the person with cancer’, ‘being involved in the person with cancer’s care, together with the medical team’, ‘balancing the needs of the person with cancer and your own needs’ and ‘adjusting to changes in the person with cancer’s body’.

## 4. Discussion

This population-based study of 84 pancreatic cancer patient–carer dyads is the first to identify that nearly two-thirds (63%) of pancreatic cancer carers reported having at least one moderate-to-high level need, the most prevalent of which were in the healthcare service and information domains. Importantly, we further identified that carers who reported more than 13 unmet needs (i.e., the highest tertile of needs vs. the lowest tertile of needs) were significantly more likely to be experiencing anxiety or depression and caring for a patient with anxiety or depression. Although the direction of the effect cannot be inferred, this nevertheless sends a strong message that we need to ensure that the basic healthcare and educational requirements of this caregiver population are met, not only to provide optimal care to carers but potentially to also reduce carers’ and patients’ psychological morbidity.

A systematic review quantifying unmet needs in carers for adults with cancers across eight studies found that 16–68% of carers had at least one unmet need [18]. The highest prevalence of 68% was reported by carers for patients with colorectal cancer two months after their diagnosis [19]; however this study combined low, moderate and high needs in their prevalence of unmet needs, whereas we combined only moderate and high needs. The prevalence of unmet needs in the other seven studies from this systematic review was lower than what we observed [20,21,22,23,24,25,26]. The variation in prevalence may be attributable to different measures and cut points used to assess unmet needs, but it also may be due to the characteristics of the patient population. Carers for people with pancreatic cancer, with which the majority of patients have an incurable disease at the outset and a significantly shorter expected survival, may respond differently to other populations in which the disease is potentially curative at diagnosis. 

Similar to a recent qualitative literature review on the needs of people caring for patients with pancreatic cancer which identified key domains as ‘clinical communication’, ‘support and briefings’ and ‘navigating health systems’ [9], we found that the healthcare and information need domains were priorities. In particular, we found that approximately a third of carers felt inadequately informed about the benefits and side-effects of treatments, the person with cancer’s prognosis and physical needs, and about financial support. We also identified that a third of carers were not feeling supported in relation to opportunities to discuss concerns with doctors, being able to be involved in care together with the medical team, help with fear about the patients’ deterioration and ensuring that there is an ongoing case manager to coordinate services. Existing quantitative studies of carers for advanced cancer patients, irrespective of the type of cancer, found that information needs were the most commonly reported unmet needs, including illness and treatment information (26–100%) and care-related information (21–100%) [27]. In addition to information needs, other domains such as physical and psychological well-being, healthcare services and spiritual and social lives were also identified [27]. 

We show that all four need domains (healthcare, information, psychological and work/social) we measured were related to patient and carer anxiety and carer depression and that all but the information domain were also significantly associated with patient depression. Furthermore, we found that the higher the number of unmet needs reported by carers, the higher the psychological morbidity both they and their patients were experiencing. A study of cancer carers in the United States found similar associations between psychosocial and medical need domains and carers’ mental health [19], and a Taiwanese study of carers for people with advanced lung cancer also found strong associations between patients’ anxiety or depression and carers having healthcare, psychological and work/social needs [28]. Both studies emphasise the importance of integrating needs assessment for carers into routine clinical care to minimise the likelihood of patients and carers experiencing poor psychological health. However, the practicalities of achieving this are complicated in a hospital setting in which staff have limited time [29] and the carer is not ‘a patient’ and so cannot directly be provided and billed for care. Such assessments may require early referral to palliative care in which person- and family-centred care are provided. 

Our finding that carers’ anxiety and depression were associated with a higher number of unmet carer needs was in line with previous reports for Icelandic carers for patients with various types of cancer [30], and our finding that patients’ anxiety and depression were associated with a higher number of unmet carer needs was in line with previous reports from a study of multiple myeloma [24]. 

Importantly, we identified the need for help with making decisions in the context of uncertainty to be the most strongly associated with carers’ anxiety or depression and that needing help with ‘accessing information about the benefits and side-effects of treatments’, ‘obtaining the best medical care for the person with cancer’ and ‘being involved in the person with cancer’s care, together with the medical team’ were significantly associated with all psychological outcomes for carers and patients. Again, this speaks to the need to actively include carers in medical consultations where possible. 

Finally, we identified that being a male carer and caring for a patient in the first four months after their diagnosis were associated with greater unmet needs. Higher needs closer to the time of diagnosis might reflect increased demands immediately after the diagnosis and during initial treatment [31]. Although the difference in needs between male and female carers found in this study was relatively small, it may have implications for future interventions. In some Western societies, it is less common for men to take on a caregiving role [32]. Men may need more support to feel comfortable and confident [33,34,35,36].

Clinical practice guidelines recommend that clinicians discuss and develop supportive care plans in consultation with patients and their carers [29]. Further, the optimal care pathways for people with pancreatic cancer state that given the poor prognosis, all patients with pancreatic cancer should be offered an early referral for a palliative care assessment as an integrated aspect of their overall oncology care [37]. Many studies have shown that palliative care interventions improve health outcomes among carers, including decreased levels of psychological distress [38]. However, it is estimated that only 28% of pancreatic cancer patients see a care coordinator or have a care plan, and less than half (45%) access palliative care [39,40]. A lack of communication about palliative care may exist when healthcare professionals fear damaging patients’ and carers’ hope [41]. Investments in programs to improve information exchange as well as shared decision making between health professionals and family carers could be useful to better meet carers’ needs [38]. 

A major strength of this study is the use of validated measures to quantitatively assess unmet needs and psychological outcomes in the largest cohort of pancreatic cancer carers to date. However, several limitations should be taken into account. Firstly, the analysis was cross-sectional, so temporality cannot be inferred; that is, we did not look at anxiety and depression at future time points due to significant missing repeated measures as participants died or became moribund. We also did not have information about pre-existing anxiety or depression to adjust for confounding. Thus, we do not know if having high unmet needs leads to poor psychological outcomes or if having pre-existing anxiety or depression results in high needs. Nevertheless, meeting needs remains important irrespective of the direction of the effect. Secondly, a higher proportion of patients in the study had a surgical resection than is expected in the general population. However, we found that the disease status in our patients was not associated with carers having high unmet needs and is thus likely to have had relatively little impact on the results. Thirdly, as the study used a representative cohort from Queensland, a state in Australia, it may limit the generalisability of the results to different populations across other states and territories, as well as countries. A further limitation is that the data were collected over 10 years ago, and changes in clinical practice (e.g., more access to cancer care coordinators and palliative care services) and the prevalence of unmet needs in carers may have occurred. Up-to-date data are necessary to inform service providers of current needs for intervention.

## 5. Conclusions

This study contributes to the understanding of the needs of people caring for patients with pancreatic cancer and the relationship between needs and anxiety and depression in both carers and patients. Although clinical practice guidelines provide evidence-based recommendations for the delivery of optimal care to cancer patients and their family members, our study found that many carers for pancreatic cancer patients report unmet needs, particularly in relation to healthcare and information. This may be due to the lack of involvement of the carer as part of the healthcare team or a lack of early referral to palliative care in which carers’ needs are assessed. Failing to meet carers’ expectations for information and involve them in the person with cancer’s care can have significant associations with adverse psychological outcomes in patients and the carers themselves. The association of carers’ needs with both carer and patient anxiety and depression provides a strong rationale for the implementation of optimal care guidelines to promote early referral to palliative care and routine assessments of supportive care needs among family carers for triage to effective interventions to manage those needs.

## Figures and Tables

**Table 1:** Demographic and clinical characteristics of participants.

Characteristics	Carers (n = 84)	Patients (n = 84)
	n (%)	n (%)
Relationship to patient		
Husband/wife/partner	68 (81.0)	
Son/daughter	10 (11.9)	
Father/mother/friend/other	6 (7.1)	
Lives with patient		
Yes	79 (94)	
No	5 (6)	
Gender		
Male	23 (27.4)	55 (65.5)
Female	61 (72.6)	29 (34.5)
Age		
≤60 years	32 (38.1)	22 (26.2)
61–70 years	31 (36.9)	33 (39.3)
≥71 years	21 (25.0)	29 (34.5)
Highest level of education		
High school or lower	48 (57.1)	37 (44.0)
College/diploma/trade certificate	21 (25)	33 (39.3)
University	15 (17.9)	14 (16.7)
Disease status		
Resection completed—curative disease		30 (35.7)
No resection—locally advanced disease		20 (23.8)
No resection—metastatic disease		30 (35.7)
Resection not attempted due to age, comorbidities or patient decline		4 (4.8)
Months post diagnosis		
≤2 months		29 (34.5)
3–4 months		40 (47.6)
5–9 months		15 (17.9)

**Table 2:** Moderate-to-high unmet needs reported by carers for pancreatic cancer patients.

Rank	Need Item	Domain	Mod–High Need	
			n	%
1	Accessing information about what the person with cancer’s physical needs are	Information	31	39
2	Having opportunities to discuss your concerns with doctors	Healthcare	29	36
3	Finding more accessible hospital parking	Other	29	36
4	Addressing fears about the person with cancer’s deterioration	Healthcare	28	35
5	Accessing information about the benefits and side-effects of treatments	Information	27	34
6	Being involved in the person with cancer’s care, together with a medical team	Healthcare	26	32
7	Finding out about financial support and government benefits	Information	26	32
8	Accessing information about the person with cancer’s prognosis	Information	24	30
9	Ensuring that there is an ongoing case manager to coordinate services	Healthcare	23	29
10	Obtaining the best medical care for the person with cancer	Healthcare	22	28
11	Balancing the needs of the person with cancer and your own needs	Psychological	22	27
12	Accessing information relevant to your needs as a carer/partner	Information	20	26
13	Feeling confident that all the doctors are talking to each other	Healthcare	21	26
14	The impact that caring for the person with cancer has had on your working life, or usual activities	Work/social	20	25
15	Accessing information about support services for carers/partners	Information	19	24
16	Adapting to changes in the person with cancer’s working life or usual activities	Work/social	19	24
17	Accessing local health care services when needed	Healthcare	19	23
18	Understanding the experience of the person with cancer	Psychological	19	23
19	Making decisions about your life in the context of uncertainty	Psychological	18	23
20	Finding meaning in the person with cancer’s illness	Psychological	18	23
21	Making sure that complaints regarding the person with cancer’s care are properly addressed	Healthcare	18	22
22	Obtaining adequate pain control for the person with cancer	Healthcare	17	21
23	Accessing information about alternative therapies	Information	15	19
24	Working through your feelings about death and dying	Psychological	15	19
25	Dealing with others not acknowledging the impact caring has on your life	Psychological	15	19
26	Adjusting to changes in the person with cancer’s body	Psychological	14	18
27	Looking after your own health, including eating and sleeping properly	Healthcare	14	17
28	Obtaining emotional support for your loved ones	Psychological	13	16
29	Communicating with family	Work/social	12	15
30	Managing concerns about the cancer returning	Psychological	12	15
31	Obtaining emotional support for yourself	Psychological	12	15
32	Coping with the person with cancer’s recovery not turning out the way you expected	Psychological	12	15
33	The impact cancer has had on your relationship with the person with cancer	Psychological	11	14
34	Exploring your spiritual beliefs	Psychological	10	13
35	Having opportunities to participate in making decisions about treatment	Information	9	12
36	Communicating with the person you are caring for	Work/social	9	11
37	Receiving more support from your family	Work/social	8	10
38	Talking to other people who have cared for someone with cancer	Work/social	8	10
39	Caring for the person with cancer on a practical level, such as with bathing, changing dressings, or giving medications	Other	6	7
40	Obtaining life and/or travel insurance for the person with cancer	Other	5	6
41	Accessing legal services	Other	5	6
42	Addressing problems with your sex life	Psychological	5	6
43	Handling the topic of cancer in social situations or at work	Work/social	3	4
44	Accessing information about potential fertility problems in the person with cancer	Other	2	3

**Table 3:** Personal and disease risk factors associated with carers having at least one moderate-to-high unmet supportive care need.

Personal and Disease Factors	Having at Least One Moderate-to-High Unmet Supportive Care Need		PR (95% CI)
	No n = 31 (36.9%)	Yes n = 53 (63.1%)	
	n (Column %)	n (Column %)	
Carer’s gender			
Male	5 (16.1)	18 (34.0)	1.36 (1.01–1.85)
Female	26 (83.9)	35 (66.0)	1
Carer’s age			
≤60 years	11 (35.5)	21 (39.6)	1.06 (0.70–1.61)
61–70 years	12 (38.7)	19 (35.8)	0.99 (0.64–1.53)
≥71 years	8 (25.8)	13 (24.5)	1
Carer’s relationship with the patient			
Husband/Wife/Partner	26 (83.9)	42 (79.2)	1
Other	5 (16.1)	11 (20.8)	1.11 (0.76–1.63)
Carers’ education level			
High school or less	21 (67.7)	27 (50.9)	1
College/diploma/trade certificate	5 (16.1)	16 (30.2)	1.35 (0.96–1.91)
University	5 (16.1)	10 (18.9)	1.19 (0.77–1.83)
Patient’s disease status			
Resection completed—curative disease	10 (32.3)	20 (40.8)	1.33 (0.86–2.07)
No resection—locally advanced disease	6 (19.4)	14 (28.6)	1.40 (0.88–2.21)
No resection—metastatic disease	15 (48.4)	15 (30.6)	1
Resection not attempted		4	
Months after the patient’s diagnosis			
≤2 months	10 (32.3)	19 (35.8)	2.46 (1.02–5.92)
3–4 months	10 (32.3)	30 (56.6)	2.81 (1.19–6.63)
5–9 months	11 (35.5)	4 (7.5)	1

**Table 4:** The association of carers’ supportive care needs with carers’ and patients’ anxiety and depression.

	n (%)	Carers’ Subclinical or Clinical Anxiety	Carers’ Subclinical or Clinical Depression	Patients’ Subclinical or Clinical Anxiety	Patients’ Subclinical or Clinical Depression
		Unadjusted PR (95% CI)	Unadjusted PR (95% CI)	Unadjusted PR (95% CI)	Unadjusted PR (95% CI)
Individual need domains					
Having at least one moderate-to-high unmet need in the healthcare service domain					
No	46 (55)	1	1	1	1
Yes	38 (45)	2.42 (1.55–3.78)	5.08 (2.12–12.20)	2.94 (1.36–6.34)	2.06 (1.07–3.95)
Having at least one moderate-to-high unmet need in the psychological/emotional domain					
No	45 (54)	1	1	1	1
Yes	39 (46)	2.55 (1.61–4.06)	3.85 (1.72–8.61)	4.38 (1.81–10.64)	1.96 (1.02–3.77)
Having at least one moderate-to-high unmet need in the information domain					
No	39 (46)	1	1	1	1
Yes	45 (54)	2.38 (1.44–3.94)	2.89 (1.29–6.46)	3.29 (1.36–8.00)	1.47 (0.77–2.83)
Having at least one moderate-to-high unmet need in the work/social domain					
No	50 (60)	1	1	1	1
Yes	34 (40)	2.42 (1.60–3.67)	3.31 (1.63–6.73)	2.06 (1.04–4.08)	2.14 (1.14–4.02)
Number of domains with least one moderate-to-high unmet need					
0	34 (40)	1	1	1	1
1–3	25 (30)	2.72 (1.39–5.33)	2.38 (0.78–7.26)	2.38 (0.78–7.26)	1.02 (0.40–2.57)
4	25 (30)	3.57 (1.90–6.70)	5.10 (1.92–13.52)	4.42 (1.63–11.95)	2.21 (1.08–4.51)
Number of moderate-to-high unmet need items reported					
Lowest tertile (0)	31 (37)	1	1	1	1
Middle tertile (1–12)	25 (30)	3.01 (1.49–6.10)	1.86 (0.59–5.88)	3.31 (0.98–11.18)	1.65 (0.66–4.14)
Highest tertile (13–35)	28 (33)	3.32 (1.67–6.60)	4.43 (1.68–11.67)	4.80 (1.52–15.10)	2.40 (1.06–5.45)

## Data Availability

The data are available upon request and with appropriate Human Research Ethics Committee approval and data transfer agreements in place.

## Supplementary Materials

The following supporting information can be downloaded at https://www.mdpi.com/article/10.3390/cancers15225307/s1, Table S1: Numbers and percentages of subclinical or clinical anxiety or depression among carers or patients; Table S2: Numbers and percentages of subclinical or clinical anxiety or depression among carers or patients by individual carer needs; Table S3: Associations between carers’ unmet needs and carers’ and patients’ subclinical or clinical anxiety or depression.
