# Supplementary material for: The Unmet Needs of Pancreatic Cancer Carers Are Associated with Anxiety and Depression in Patients and Carers
Source: Cancers (Basel). 2023 Nov 7;15(22):5307. doi: 10.3390/cancers15225307 (PMC10670364; doi:10.3390/cancers15225307)
Supplement: Supplementary file 1 [file cancers-15-05307-s001.zip › cancers-2616520-supplementary.pdf]

Supplementary Material: The Unmet Needs of Pancreatic Cancer Carers Are Associated with Anxiety and Depression in Patients and Carers

Supplementary Table S1. Numbers and percentages of subclinical or clinical anxiety or depression among carers or patients.

	**Carers' anxiety**		**Carers' depression**		**Patients' anxiety**		**Patients'depression**	
	**No (N = 39)**	**Yes (N = 45)**	**No (N = 58)**	**Yes (N = 26)**	**No (N = 60)**	**Yes (N = 24)**	**No (N = 57)**	**Yes (N = 27)**
	**n (%)**	**n (%)**	**n (%)**	**n (%)**	**n (%)**	**n (%)**	**n (%)**	**n(%)**
**Carers having at least one moderate-to-high unmet supportive care need**								
No	24 (77)	7 (23)	27 (87)	4 (13)	28 (90)	3 (10)	25 (81)	6 (19)
Yes	15 (28)	38 (72)	31 (58)	22 (42)	32 (60)	21 (40)	32 (60)	21 (40)
**Individual need domain**								
Carers having at least one moderate-to-high unmet need in the healthcare service domain								
No	31 (67)	15 (33)	41 (89)	5 (11)	39 (85)	7 (15)	36 (78)	10 (22)
Yes	8 (21)	30 (79)	17 (45)	21 (55)	21 (55)	17 (45)	21 (55)	17 (45)
Carers having at least one moderate-to-high unmet need in the psychological/emotional domain								
No	31 (69)	14 (31)	39 (87)	6 (13)	40 (89)	5 (11)	35 (78)	10 (22)
Yes	8 (21)	31 (79)	19 (49)	20 (51)	20 (51)	19 (49)	22 (56)	17 (44)
Carers having at least one moderate-to-high unmet need in the information domain								
No	27 (69)	12 (31)	33 (85)	6 (15)	34 (87)	5 (13)	29 (74)	10 (26)
Yes	12 (27)	33 (73)	25 (56)	20 (44)	26 (58)	19 (42)	28 (62)	17 (38)
Carers having at least one moderate-to-high unmet need in the work/social domains								
No	33 (66)	17 (34)	42 (84)	8 (16)	40 (80)	10 (20)	39 (78)	11 (22)
Yes	6 (18)	28 (82)	16 (47)	18 (53)	20 (59)	14 (41)	18 (53)	16 (47)
**Number of domains with least one moderate-to-high unmet need**								
0	26 (76)	8 (24)	30 (88)	4 (12)	30 (88)	4 (12)	26 (76)	8 (24)
1	4 (67)	2 (33)	5 (83)	1 (17)	5 (83)	1 (17)	5 (83)	1 (17)
2	1 (14)	6 (86)	7 (100)	0 (0)	5 (71)	2 (29)	6 (86)	1 (14)
3	4 (33)	8 (67)	6 (50)	6 (50)	8 (67)	4 (33)	8 (67)	4 (33)
4	4 (16)	21 (84)	10 (40)	15 (60)	12 (48)	13 (52)	12 (48)	13 (52)
**Number of moderate to high need items reported**								
Lowest tertile (0 needs)	24 (77)	7 (23)	27 (87)	4 (13)	28 (90)	3 (9.7)	25 (81)	6 (19)
Middle tertile (1-12 needs)	8 (32)	17 (68)	19 (76)	6 (24)	17 (68)	8 (32)	17 (68)	8 (32)
Highest tertile (13-35 needs)	7 (25)	21 (75)	12 (43)	16 (57)	15 (54)	13 (46)	15 (54)	13 (46)

Supplementary Table S2. Numbers and percentages of subclinical or clinical anxiety or depression among carers or patients by individual carer needs

	**Carers' anxiety**		**Carers' depression**		**Patients' anxiety**		**Patients' depression**	
	**No (N = 39)**	**Yes (N = 45)**	**No (N = 58)**	**Yes (N = 26)**	**No (N = 60)**	**Yes (N = 24)**	**No (N = 57)**	**Yes (N = 27)**
	**n (%)**	**n (%)**	**n (%)**	**n (%)**	**n (%)**	**n (%)**	**n (%)**	**n(%)**
Accessing information relevant to your needs as a carer/partner								
No	33 (57)	25 (43)	48 (83)	10 (17)	44 (76)	14 (24)	39 (67)	19 (33)
Yes	3 (15)	17 (85)	7 (35)	13 (65)	12 (60)	8 (40)	14 (70)	6 (30)
Accessing information about the person with cancer’s prognosis, or likely outcome								
No	33 (60)	22 (40)	46 (84)	9 (16)	41 (75)	14 (25)	39 (71)	16 (29)
Yes	3 (12)	21 (88)	9 (38)	15 (62)	16 (67)	8 (33)	16 (67)	8 (33)
Accessing information about support services for carers/partners of people with cancer								
No	32 (53)	28 (47)	49 (82)	11 (18)	44 (73)	16 (27)	40 (67)	20 (33)
Yes	4 (21)	15 (79)	6 (32)	13 (68)	12 (63)	7 (37)	14 (74)	5 (26)
Accessing information about alternative therapies								
No	30 (48)	33 (52)	48 (76)	15 (24)	46 (73)	17 (27)	44 (70)	19 (30)
Yes	5 (33)	10 (67)	6 (40)	9 (60)	9 (60)	6 (40)	9 (60)	6 (40)
Accessing information on what the person with cancer’s physical needs are likely to be								
No	28 (58)	20 (42)	42 (88)	6 (12)	39 (81)	9 (19)	36 (75)	12 (25)
Yes	8 (26)	23 (74)	13 (42)	18 (58)	17 (55)	14 (45)	18 (58)	13 (42)
Accessing information about the benefits and side-effects of treatments								
No	31 (58)	22 (42)	44 (83)	9 (17)	42 (79)	11 (21)	41 (77)	12 (23)
Yes	6 (22)	21 (78)	12 (44)	15 (56)	15 (56)	12 (44)	14 (52)	13 (48)
Obtaining the best medical care for the person with cancer								
No	32 (56)	25 (44)	47 (82)	10 (18)	46 (81)	11 (19)	43 (75)	14 (25)
Yes	5 (23)	17 (77)	9 (41)	13 (59)	11 (50)	11 (50)	11 (50)	11 (50)
Accessing local health care services when needed								
No	34 (55)	28 (45)	49 (79)	13 (21)	47 (76)	15 (24)	44 (71)	18 (29)
Yes	3 (16)	16 (84)	7 (37)	12 (63)	10 (53)	9 (47)	11 (58)	8 (42)
Being involved in the person with cancer’s care, together with the medical team								
No	31 (56)	24 (44)	45 (82)	10 (18)	43 (78)	12 (22)	42 (76)	13 (24)
Yes	6 (23)	20 (77)	11 (42)	15 (58)	14 (54)	12 (46)	13 (50)	13 (50)
Having opportunities to discuss your concerns with the doctors								
No	30 (58)	22 (42)	42 (81)	10 (19)	40 (77)	12 (23)	40 (77)	12 (23)
Yes	7 (24)	22 (76)	14 (48)	15 (52)	17 (59)	12 (41)	15 (52)	14 (48)
Feeling confident that all the doctors are talking to each other to coordinate the person with cancer’s care								
No	33 (56)	26 (44)	48 (81)	11 (19)	45 (76)	14 (24)	42 (71)	17 (29)
Yes	4 (19)	17 (81)	8 (38)	13 (62)	12 (57)	9 (43)	12 (57)	9 (43)
Ensuring there is an ongoing case manager to coordinate services for the person with cancer								
No	33 (58)	24 (42)	46 (81)	11 (19)	44 (77)	13 (23)	42 (74)	15 (26)
Yes	4 (17)	19 (83)	10 (43)	13 (57)	13 (57)	10 (43)	13 (57)	10 (43)
Making sure complaints regarding the person with cancer’s care are properly addressed								
No	33 (52)	30 (48)	49 (78)	14 (22)	46 (73)	17 (27)	45 (71)	18 (29)
Yes	4 (22)	14 (78)	7 (39)	11 (61)	11 (61)	7 (39)	10 (56)	8 (44)
Reducing stress in the person with cancer’s life								
No	33 (49)	34 (51)	49 (73)	18 (27)	46 (69)	21 (31)	45 (67)	22 (33)
Yes	4 (29)	10 (71)	7 (50)	7 (50)	11 (79)	3 (21)	10 (71)	4 (29)
Looking after your own health, including eating and sleeping properly								
No	10 (71)	4 (29)	12 (86)	2 (14)	13 (93)	1 (7.1)	12 (86)	2 (14)
Yes	4 (40)	6 (60)	4 (40)	6 (60)	5 (50)	5 (50)	5 (50)	5 (50)
Obtaining adequate pain control for the person with cancer								
No	33 (52)	30 (48)	48 (76)	15 (24)	47 (75)	16 (25)	45 (71)	18 (29)

	Carers' anxiety		Carers' depression		Patients' anxiety		Patients' depression	
	No (N = 39)	Yes (N = 45)	No (N = 58)	Yes (N = 26)	No (N = 60)	Yes (N = 24)	No (N = 57)	Yes (N = 27)
	n (%)	n (%)	n (%)	n (%)	n (%)	n (%)	n (%)	n(%)
Yes	4 (24)	13 (76)	7 (41)	10 (59)	9 (53)	8 (47)	9 (53)	8 (47)
Addressing fears about the person with cancer's physical or mental deterioration								
No	31 (61)	20 (39)	44 (86)	7 (14)	41 (80)	10 (20)	38 (75)	13 (25)
Yes	6 (21)	22 (79)	11 (39)	17 (61)	15 (54)	13 (46)	15 (54)	13 (46)
Accessing information about the potential fertility problems in the person with cancer								
No	37 (48)	40 (52)	54 (70)	23 (30)	55 (71)	22 (29)	53 (69)	24 (31)
Yes	0 (0)	2 (100)	1 (50)	1 (50)	1 (50)	1 (50)	1 (50)	1 (50)
Caring for person with cancer on a practical level, such as with bathing, changing dressings or giving medications								
No	34 (45)	41 (55)	52 (69)	23 (31)	54 (72)	21 (28)	53 (71)	22 (29)
Yes	3 (50)	3 (50)	4 (67)	2 (33)	3 (50)	3 (50)	2 (33)	4 (67)
Finding more accessible hospital parking								
No	28 (54)	24 (46)	39 (75)	13 (25)	40 (77)	12 (23)	40 (77)	12 (23)
Yes	9 (31)	20 (69)	17 (59)	12 (41)	17 (59)	12 (41)	15 (52)	14 (48)
Adapting to changes to the person with cancer's working life, or usual activities								
No	33 (54)	28 (46)	46 (75)	15 (25)	44 (72)	17 (28)	45 (74)	16 (26)
Yes	4 (21)	15 (79)	9 (47)	10 (53)	12 (63)	7 (37)	9 (47)	10 (53)
The impact that caring for the person with cancer has had on your working life, or usual activities								
No	35 (57)	26 (43)	48 (79)	13 (21)	45 (74)	16 (26)	43 (70)	18 (30)
Yes	3 (15)	17 (85)	8 (40)	12 (60)	12 (60)	8 (40)	11 (55)	9 (45)
Finding out about financial support and government benefits for you and/or the person with cancer								
No	30 (56)	24 (44)	43 (80)	11 (20)	42 (78)	12 (22)	38 (70)	16 (30)
Yes	8 (31)	18 (69)	13 (50)	13 (50)	15 (58)	11 (42)	16 (62)	10 (38)
Obtaining life and/or travel insurance for the person with cancer								
No	36 (49)	38 (51)	53 (72)	21 (28)	53 (72)	21 (28)	50 (68)	24 (32)
Yes	1 (20)	4 (80)	2 (40)	3 (60)	3 (60)	2 (40)	3 (60)	2 (40)
Accessing legal services								
No	37 (49)	38 (51)	55 (73)	20 (27)	53 (71)	22 (29)	51 (68)	24 (32)
Yes	1 (20)	4 (80)	1 (20)	4 (80)	4 (80)	1 (20)	3 (60)	2 (40)
Communicating with the person you are caring for								
No	37 (51)	36 (49)	53 (73)	20 (27)	54 (74)	19 (26)	51 (70)	22 (30)
Yes	1 (11)	8 (89)	4 (44)	5 (56)	4 (44)	5 (56)	4 (44)	5 (56)
Communicating with the family								
No	36 (51)	34 (49)	52 (74)	18 (26)	52 (74)	18 (26)	51 (73)	19 (27)
Yes	2 (17)	10 (83)	5 (42)	7 (58)	6 (50)	6 (50)	4 (33)	8 (67)
Getting more support from your family								
No	38 (51)	36 (49)	53 (72)	21 (28)	54 (73)	20 (27)	52 (70)	22 (30)
Yes	0 (0)	8 (100)	4 (50)	4 (50)	4 (50)	4 (50)	3 (38)	5 (62)
Talking to other people who have cared for someone with cancer								
No	37 (51)	35 (49)	56 (78)	16 (22)	52 (72)	20 (28)	49 (68)	23 (32)
Yes	1 (12)	7 (88)	0 (0)	8 (100)	5 (62)	3 (38)	5 (62)	3 (38)
Handling the topic of cancer in social situations or at work								
No	38 (49)	39 (51)	56 (73)	21 (27)	56 (73)	21 (27)	53 (69)	24 (31)
Yes	0 (0)	3 (100)	0 (0)	3 (100)	1 (33)	2 (67)	1 (33)	2 (67)
Managing concerns about the cancer coming back								
No	37 (55)	30 (45)	52 (78)	15 (22)	48 (72)	19 (28)	45 (67)	22 (33)
Yes	1 (8)	11 (92)	3 (25)	9 (75)	8 (67)	4 (33)	8 (67)	4 (33)
The impact that cancer has had on your relationship with the person with cancer								
No	36 (51)	34 (49)	53 (76)	17 (24)	53 (76)	17 (24)	50 (71)	20 (29)
Yes	2 (18)	9 (82)	4 (36)	7 (64)	5 (45)	6 (55)	5 (45)	6 (55)
Understanding the experience of the person with cancer								

	Carers' anxiety		Carers' depression		Patients' anxiety		Patients' depression	
	No (N = 39)	Yes (N = 45)	No (N = 58)	Yes (N = 26)	No (N = 60)	Yes (N = 24)	No (N = 57)	Yes (N = 27)
	n (%)	n (%)	n (%)	n (%)	n (%)	n (%)	n (%)	n(%)
No	36 (58)	26 (42)	48 (77)	14 (23)	48 (77)	14 (23)	44 (71)	18 (29)
Yes	2 (11)	17 (89)	9 (47)	10 (53)	10 (53)	9 (47)	11 (58)	8 (42)
Balancing the needs of the person with cancer and your own needs								
No	33 (56)	26 (44)	47 (80)	12 (20)	48 (81)	11 (19)	44 (75)	15 (25)
Yes	5 (23)	17 (77)	10 (45)	12 (55)	10 (45)	12 (55)	11 (50)	11 (50)
Adjusting to changes in the person with cancer's body								
No	35 (53)	31 (47)	51 (77)	15 (23)	52 (79)	14 (21)	50 (76)	16 (24)
Yes	3 (21)	11 (79)	5 (36)	9 (64)	5 (36)	9 (64)	4 (29)	10 (71)
Addressing problems with your sex life								
No	34 (46)	40 (54)	51 (69)	23 (31)	54 (73)	20 (27)	51 (69)	23 (31)
Yes	3 (60)	2 (40)	4 (80)	1 (20)	2 (40)	3 (60)	2 (40)	3 (60)
Getting emotional support for yourself								
No	36 (52)	33 (48)	52 (75)	17 (25)	50 (72)	19 (28)	47 (68)	22 (32)
Yes	2 (17)	10 (83)	4 (33)	8 (67)	7 (58)	5 (42)	7 (58)	5 (42)
Getting emotional support for your loved ones								
No	35 (51)	33 (49)	50 (74)	18 (26)	49 (72)	19 (28)	48 (71)	20 (29)
Yes	3 (23)	10 (77)	6 (46)	7 (54)	8 (62)	5 (38)	6 (46)	7 (54)
Working through your feelings about death and dying								
No	36 (55)	30 (45)	52 (79)	14 (21)	50 (76)	16 (24)	46 (70)	20 (30)
Yes	2 (13)	13 (87)	4 (27)	11 (73)	7 (47)	8 (53)	8 (53)	7 (47)
Dealing with others not acknowledging the impact on your life of caring for a person with cancer								
No	37 (56)	29 (44)	50 (76)	16 (24)	48 (73)	18 (27)	44 (67)	22 (33)
Yes	1 (7)	14 (93)	6 (40)	9 (60)	9 (60)	6 (40)	10 (67)	5 (33)
Coping with the person with cancer's recovery not turning out the way you expected								
No	37 (55)	30 (45)	51 (76)	16 (24)	51 (76)	16 (24)	45 (67)	22 (33)
Yes	1 (3)	11 (27)	5 (8.9)	7 (30)	5 (8.9)	7 (30)	8 (15)	4 (15)
Making decisions about your life in the context of uncertainty								
No	36 (60)	24 (40)	46 (77)	14 (23)	44 (73)	16 (27)	41 (68)	19 (32)
Yes	2 (11)	16 (89)	9 (50)	9 (50)	11 (61)	7 (39)	11 (61)	7 (39)
Exploring your spiritual beliefs								
No	36 (52)	33 (48)	51 (74)	18 (26)	48 (70)	21 (30)	48 (70)	21 (30)
Yes	1 (10)	9 (90)	4 (40)	6 (60)	8 (80)	2 (20)	5 (50)	5 (50)
Finding meaning in the person with cancer's illness								
No	35 (59)	24 (41)	46 (78)	13 (22)	41 (69)	18 (31)	40 (68)	19 (32)
Yes	1 (6)	17 (94)	7 (39)	11 (61)	13 (72)	5 (28)	11 (61)	7 (39)
Having opportunities to participate in decision making about the person with cancer's treatment								
No	35 (51)	33 (49)	50 (74)	18 (26)	51 (75)	17 (25)	48 (71)	20 (29)
Yes	1 (11)	8 (89)	4 (44)	5 (56)	4 (44)	5 (56)	4 (44)	5 (56)

Supplementary Table S3. Associations between carers' unmet needs and carers' and patients' subclinical or clinical anxiety or depression.

	Carers' anxiety	Carers' depression	Patients' anxiety	Patients' depression
	Unadjusted PR[a] (95% CI)[b]	Unadjusted PR (95% CI)	Unadjusted PR (95% CI)	Unadjusted PR (95% CI)
**Having at least one moderate-to-high unmet supportive care need**				
Yes vs. No	3.18 (1.62-6.23)	3.22 (1.22-8.48)	4.09 (1.33-12.62)	2.05 (0.93-4.52)
**Individual need item**				
Accessing information relevant to your needs as a carer/partner				
Yes vs. No	1.97 (1.39-2.79)	3.77 (1.97-7.22)	1.66 (0.82-3.35)	0.92 (0.43-1.97)
Accessing information about the person with cancer's prognosis, or likely outcome				
Yes vs. No	2.19 (1.53-3.13)	3.82 (1.95-7.49)	1.31 (0.63-2.70)	1.15 (0.57-2.31)
Accessing information about support services for carers/partners of people with cancer				
Yes vs. No	1.69 (1.18-2.42)	3.73 (2.02-6.90)	1.38 (0.67-2.85)	0.79 (0.34-1.82)
Accessing information about alternative therapies				
Yes vs. No	1.27 (0.83-1.95)	2.52 (1.38-4.61)	1.48 (0.71-3.11)	1.33 (0.64-2.74)
Accessing information on what the person with cancer's physical needs are likely to be				
Yes vs. No	1.78 (1.20-2.64)	4.65 (2.07-10.40)	2.41 (1.19-4.88)	1.68 (0.88-3.19)
Accessing information about the benefits and side-effects of treatments				
Yes vs. No	1.87 (1.28-2.73)	3.27 (1.65-6.49)	2.14 (1.09-4.20)	2.13 (1.13-4.01)
Obtaining the best medical care for the person with cancer				
Yes vs. No	1.76 (1.22-2.55)	3.37 (1.74-6.53)	2.59 (1.32-5.09)	2.04 (1.10-3.78)
Accessing local health care services when needed				
Yes vs. No	1.86 (1.33-2.61)	3.01 (1.66-5.45)	1.96 (1.03-3.74)	1.45 (0.75-2.79)
Being involved in the person with cancer's care, together with the medical team				
Yes vs. No	1.76 (1.22-2.54)	3.17 (1.66-6.08)	2.12 (1.10-4.05)	2.12 (1.15-3.90)
Having opportunities to discuss your concerns with the doctors				
Yes vs. No	1.79 (1.23-2.62)	2.69 (1.39-5.20)	1.79 (0.93-3.46)	2.09 (1.12-3.90)
Feeling confident that all the doctors are talking to each other to coordinate the person with cancer's care				
Yes vs. No	1.84 (1.29-2.62)	3.32 (1.77-6.23)	1.81 (0.92-3.54)	1.49 (0.79-2.81)
Ensuring there is an ongoing case manager to coordinate services for the person with cancer				
Yes vs. No	1.96 (1.37-2.81)	2.93 (1.54-5.56)	1.91 (0.98-3.72)	1.65 (0.87-3.12)
Making sure complaints regarding the person with cancer's care are properly addressed				
Yes vs. No	1.63 (1.14-2.34)	2.75 (1.52-4.97)	1.44 (0.71-2.92)	1.56 (0.81-2.97)
Reducing stress in the person with cancer's life				
Yes vs. No	1.41 (0.94-2.11)	1.86 (0.97-3.59)	0.68 (0.24-1.98)	0.87 (0.36-2.13)
Looking after your own health, including eating and sleeping properly				
Yes vs. No	2.10 (0.80-5.54)	4.20 (1.06-16.68)	7.00 (0.96-51.10)	3.50 (0.84-14.55)
Obtaining adequate pain control for the person with cancer				
Yes vs. No	1.61 (1.11-2.32)	2.47 (1.36-4.48)	1.85 (0.96-3.58)	1.65 (0.87-3.12)
Addressing fears about the person with cancer's physical or mental deterioration				
Yes vs. No	2.00 (1.35-2.97)	4.42 (2.09-9.36)	2.37 (1.20-4.69)	1.82 (0.98-3.37)
Accessing information about the potential fertility problems in the person with cancer				
Yes vs. No	1.92 (1.55-2.39)	1.67 (0.40-6.98)	1.75 (0.42-7.31)	1.60 (0.39-6.67)
Caring for the person with cancer on a practical level, such as with bathing, changing dressings or giving medications				
Yes vs. No	0.91 (0.40-2.09)	1.09 (0.33-3.54)	1.79 (0.74-4.30)	2.27 (1.17-4.42)
Finding more accessible hospital parking				
Yes vs. No	1.49 (1.02-2.19)	1.66 (0.87-3.14)	1.79 (0.93-3.46)	2.09 (1.12-3.90)
Adapting to changes to the person with cancer's working life, or usual activities				
Yes vs. No	1.72 (1.20-2.46)	2.14 (1.16-3.95)	1.32 (0.65-2.70)	2.01 (1.10-3.65)
The impact that caring for the person with cancer has had on your working life, or usual activities				
Yes vs. No	1.99 (1.41-2.81)	2.82 (1.54-5.13)	1.52 (0.77-3.02)	1.52 (0.82-2.84)
Finding out about financial support and government benefits for you and/or the person with cancer				
Yes vs. No	1.56 (1.05-2.31)	2.45 (1.28-4.71)	1.90 (0.97-3.72)	1.30 (0.69-2.45)

	Carers' anxiety	Carers' depression	Patients' anxiety	Patients' depression
	**Unadjusted PR[a] (95% CI)[b]**	**Unadjusted PR (95% CI)**	**Unadjusted PR (95% CI)**	**Unadjusted PR (95% CI)**
Obtaining life and/or travel insurance for the person with cancer				
Yes vs. No	1.56 (0.95-2.55)	2.11 (0.95-4.71)	1.41 (0.45-4.38)	1.23 (0.40-3.79)
Accessing legal services				
Yes vs. No	1.58 (0.97-2.58)	3.00 (1.68-5.34)	0.68 (0.11-4.08)	1.25 (0.41-3.84)
Communicating with the person you are caring for				
Yes vs. No	1.80 (1.30-2.50)	2.03 (1.01-4.06)	2.13 (1.06-4.30)	1.84 (0.93-3.64)
Communicating with the family				
Yes vs. No	1.72 (1.21-2.43)	2.27 (1.22-4.23)	1.94 (0.97-3.88)	2.46 (1.41-4.28)
Getting more support from your family				
Yes vs. No	2.06 (1.63-2.60)	1.76 (0.81-3.85)	1.85 (0.84-4.07)	2.10 (1.11-3.99)
Talking to other people who have cared for someone with cancer				
Yes vs. No	1.80 (1.26-2.56)	4.50 (2.92-6.93)	1.35 (0.51-3.56)	1.17 (0.45-3.05)
Handling the topic of cancer in social situations or at work				
Yes vs. No	1.97 (1.58-2.46)	3.67 (2.55-5.28)	2.44 (1.01-5.89)	2.14 (0.90-5.09)
Managing concerns about the cancer coming back				
Yes vs. No	2.05 (1.49-2.81)	3.35 (1.93-5.82)	1.18 (0.48-2.85)	1.02 (0.43-2.42)
The impact that cancer has had on your relationship with the person with cancer				
Yes vs. No	1.68 (1.17-2.43)	2.62 (1.43-4.82)	2.25 (1.14-4.43)	1.91 (0.99-3.67)
Understanding the experience of the person with cancer				
Yes vs. No	2.13 (1.53-2.97)	2.33 (1.24-4.37)	2.10 (1.08-4.06)	1.45 (0.75-2.79)
Balancing the needs of the person with cancer and your own needs				
Yes vs. No	1.75 (1.22-2.53)	2.68 (1.42-5.05)	2.93 (1.52-5.63)	1.97 (1.07-3.60)
Adjusting to changes in the person with cancer's body				
Yes vs. No	1.67 (1.15-2.43)	2.83 (1.56-5.11)	3.03 (1.65-5.56)	2.95 (1.72-5.06)
Addressing problems with your sex life				
Yes vs. No	0.74 (0.25-2.21)	0.64 (0.11-3.84)	2.22 (0.99-4.98)	1.93 (0.87-4.26)
Getting emotional support for yourself				
Yes vs. No	1.74 (1.22-2.48)	2.71 (1.52-4.81)	1.51 (0.70-3.27)	1.31 (0.62-2.78)
Getting emotional support for your loved ones				
Yes vs. No	1.59 (1.08-2.33)	2.03 (1.07-3.86)	1.38 (0.63-3.02)	1.83 (0.98-3.42)
Working through your feelings about death and dying				
Yes vs. No	1.91 (1.37-2.65)	3.46 (1.98-6.03)	2.20 (1.16-4.16)	1.54 (0.80-2.96)
Dealing with others not acknowledging the impact on your life of caring for a person with cancer				
Yes vs. No	2.12 (1.57-2.88)	2.47 (1.37-4.48)	1.47 (0.70-3.06)	1.00 (0.45-2.21)
Coping with the person with cancer's recovery not turning out the way you expected				
Yes vs. No	2.05 (1.49-2.81)	2.44 (1.29-4.64)	2.44 (1.29-4.64)	1.02 (0.43-2.42)
Making decisions about your life in the context of uncertainty				
Yes vs. No	2.22 (1.57-3.15)	2.14 (1.12-4.11)	1.46 (0.71-2.98)	1.23 (0.62-2.44)
Exploring your spiritual beliefs				
Yes vs. No	1.88 (1.36-2.60)	2.30 (1.21-4.38)	0.66 (0.18-2.39)	1.64 (0.80-3.36)
Finding meaning in the person with cancer's illness				
Yes vs. No	2.32 (1.67-3.22)	2.77 (1.51-5.08)	0.91 (0.39-2.11)	1.21 (0.61-2.40)
Having opportunities to participate in decision making about the person with cancer's treatment				
Yes vs. No	1.83 (1.31-2.56)	2.10 (1.04-4.25)	2.22 (1.09-4.54)	1.89 (0.95-3.77)

[a] Prevalence Ratio; [b] 95% Confidence Interval.
